# Preoperative platelet to lymphocyte ratio is a valuable prognostic biomarker in patients with colorectal cancer

**DOI:** 10.18632/oncotarget.8334

**Published:** 2016-03-24

**Authors:** Jie You, Gui-Qi Zhu, Linka Xie, Wen-Yue Liu, Liang Shi, Ou-Chen Wang, Zong-Hai Huang, Martin Braddock, Gui-Long Guo, Ming-Hua Zheng

**Affiliations:** ^1^ Department of Oncological Surgery, The First Affiliated Hospital of Wenzhou Medical University, Wenzhou 325000, China; ^2^ Department of General Surgery, Zhujiang Hospital, Southern Medical University, Guangzhou 510282, China; ^3^ Department of Infection and Liver Diseases, Liver Research Center, The First Affiliated Hospital of Wenzhou Medical University, Wenzhou 325000, China; ^4^ School of The First Clinical Medical Sciences, Wenzhou Medical University, Wenzhou 325000, China; ^5^ Cancer Center of Union Hospital, Tongji Medical College, Huazhong University of Science and Technology, Wuhan 430022, China; ^6^ Department of Endocrinology, The First Affiliated Hospital of Wenzhou Medical University, Wenzhou 325000, China; ^7^ Department of Laboratory Medicine, The First Affiliated Hospital of Wenzhou Medical University, Wenzhou 325000, China; ^8^ Global Medicines Development, AstraZeneca R & D, Alderley Park, United Kingdom; ^9^ Institute of Hepatology, Wenzhou Medical University, Wenzhou 325000, China

**Keywords:** colorectal cancer, platelet to lymphocyte ratio, overall survival, disease-free survival, prognostic biomarker

## Abstract

**Objectives:**

Recent studies suggest that an elevated preoperative platelet to lymphocyte ratio (PLR) may be considered a poor prognostic biomarker in patients with colorectal cancer (CRC). The aim of this study was to evaluate the prognostic impact of PLR in patients with CRC.

**Methods:**

We enrolled 1314 patients who underwent surgery for CRC between 2005 and 2011. Preoperative PLR level was stratified into quintiles for Kaplan-Meier analysis and multivariable Cox proportional hazard regression models.

**Results:**

Higher PLR quintiles were significantly associated with poorer overall survival (*P* = 0.002). Multivariate analysis showed that PLR was an independent risk factor for overall survival (OS) (*P* = 0.034). Patients in PLR quintile 5 had lower overall survival than in quintile 1 (hazard ratio (HR) = 1.701, 95% confidence interval (CI): 1.267–2.282, *P* < 0.001). Although patients in PLR quintile 5 had significantly lower disease-free survival (DFS) than in quintile 1 (HR = 1.522, 95% CI: 1.114–2.080, *P* = 0.008), this association was not significant after multivariable adjustment (*P* = 0.075). In the subgroup analysis, PLR remained an independent factor in terms of advanced tumor stage (III, IV), male sex, carcinoembryonic antigen (≤ 5 ng/ml), age (> 65 years) and body mass index (≤ 25) (*P* < 0.05 for all measurements). The results remained unchanged when the PLR was analyzed as a dichotomous variable by applying different cut-off values of 150, 185, 220.

**Conclusions:**

Elevated preoperative PLR was independently associated with an increased risk of mortality in patients with CRC. The utility of PLR may help to improve prognostic predictors.

## INTRODUCTION

Colorectal cancer (CRC) is the third malignant neoplasm in the world and more than 600,000 people die from this disease each year [1]. On the whole, the 5-year overall survival (OS) for patients with CRC ranged from 51% to 67%, due to recurrence and metastasis of CRC [2, 3]. Conventionally, some prognostic factors, such as tumor TNM stage, cell differentiation grade and vascular invasion have been widely utilized as predictors for the prognosis of CRC. However, the survival time varies widely even in patients with the same TNM stage and tumor differentiation grade. Therefore an urgent need remains to identify optimal biomarkers that can predict progression and prognosis of the disease as complementary tools to intervention.

Recently, a number of studies have provided to evidence in support of the concept that the host inflammatory response is associated with the development and progression of cancer [4–6] and moreover, with a poor outcome independent of the tumor stage [7, 8]. Systemic inflammation can be assessed by means of peripheral blood markers such as serum white blood cells, neutrophils, lymphocyte and platelet and acute-phase proteins. Platelet to lymphocyte ratio (PLR) has been reported to be associated with poor prognosis in different tumor types, including CRC [9–14]. Nonetheless, results from several studies investigating the relationship between the PLR and the prognosis of patients with CRC remain inconsistent [15–18].

The primary objective of this study was to investigate the prognostic impact of the preoperative PLR on the survival in CRC patients and further validate the results of previous studies within a large cohort of CRC patients using different threshold values.

## RESULTS

### Baseline characteristics

Demographic and clinical characteristics of patients with CRC are listed in Table 1. There were 1314 eligible patients with available preoperative PLR levels. The mean age of patients was 66 ± 12.6 years, and the majority were male (59.7%). 697 patients (53.0%) were confirmed as presenting with rectal cancer. The majority of tumors exhibited moderate histological differentiation (70.2%). At initial diagnosis, 16.0% of the CRC patients presented with stage I, followed by 38.3% with stage II, 37.7% with stage III, and 8.1% with stage IV.

**Table 1:** Characteristics of CRC patients treated by surgical resection according to PLR quintile

Characteristic	All patients *N* (%)	PLR quintiles					*P* value
		Quintile 1 PLR ≤ 100 *N* = 297	Quintile 2 100 < PLR ≤ 120 *N* = 205	Quintile 3 120 < PLR ≤ 160 *N* = 296	Quintile 4 160 < PLR ≤ 220 *N* = 256	Quintile 5 PLR > 220 *N* = 260	
Median PLR	169.1	79.4	110.1	139.3	186.5	334.9	
Age (mean ± SD)	66.0 ± 12.6	66.7 ± 12.0	66.2 ± 11.5	66.4 ± 12.5	65.3 ± 13.2	66.5 ± 13.7	0.655
Gender							0.959
Male, *n* (%)	785 (59.7%)	178 (59.9%)	124 (60.5%)	181 (61.1%)	149 (58.2%)	153 (58.8%)	
Female, *n* (%)	529 (40.3%)	119 (40.1%)	81 (39.5%)	115 (38.9%)	107 (41.8%)	107 (41.2%)	
BMI (kg/m2)	21.9 ± 3.4	22.2 ± 3.1	22.1 ± 3.4	22.1 ± 3.4	21.8 ± 3.6	21.1 ± 3.1	< 0.001
Obesity, *n* (%)	185 (14.1%)	46 (15.5%)	37 (18.0%)	44 (14.9%)	33 (12.9%)	25 (9.6%)	0.096
Hypertension, *n* (%)	369 (28.1%)	87 (29.3%)	54 (26.3%)	94 (31.8%)	73 (28.5%)	61 (23.5%)	0.260
DM, *n* (%)	128 (9.7%)	31 (10.4%)	16 (7.8%)	30 (10.1%)	24 (9.4%)	27 (10.4%)	0.870
TNM Staging							0.008
Stage I, *n* (%)	210 (16.0%)	59 (19.9%)	42 (20.5%)	42 (14.2%)	39 (15.2%)	28 (10.8%)	
Stage II, *n* (%)	503 (38.3%)	115 (38.7%)	63 (30.7%)	124 (41.9%)	93 (36.3%)	108 (41.5%)	
Stage III, *n* (%)	495 (37.7%)	104 (35.0%)	90 (43.9%)	105 (35.5%)	102 (39.8%)	94 (36.2%)	
Stage IV, *n* (%)	106 (8.1%)	19 (6.4%)	10 (4.9%)	25 (8.4%)	22 (8.6%)	30 (11.5%)	
Histological differentiation							0.447
Well, *n* (%)	43 (3.3%)	13 (4.4%)	6 (2.9%)	11 (3.7%)	5 (2.0%)	8 (3.1%)	
Moderately, *n* (%)	923 (70.2%)	218 (73.4%)	140 (68.3%)	208 (70.3%)	184 (71.9%)	173 (66.5%)	
Poorly, *n* (%)	348 (26.5%)	66 (22.2%)	59 (28.8%)	77 (26.0%)	67 (26.2%)	79 (30.4%)	
Vascular invasion, *n* (%)	185 (14.1%)	29 (9.8%)	23 (11.2%)	46 (15.5%)	48 (18.8%)	39 (15.0%)	0.024
Location							< 0.001
Right side, *n* (%)	208 (15.8%)	32 (10.8%)	18 (8.8%)	44 (14.9%)	44 (17.2%)	70 (26.9%)	
Sigmoid, *n* (%)	232 (17.7%)	48 (16.2%)	41 (20.0%)	56 (18.9%)	45 (17.6%)	42 (16.2%)	
Rectal, *n* (%)	697 (53.0%)	183 (61.6%)	129 (62.9%)	164 (55.4%)	125 (48.8%)	96 (36.9%)	
CEA (ng/ml)	29.8 ± 146.7	24.1 ± 136.7	35.6 ± 170.8	30.9 ± 111.7	28.4 ± 173.4	31.8 ± 144.6	0.938
Creatinine (μmol/L)	68.0 ± 33.3	68.2 ± 31.2	68.9 ± 28.8	70.9 ± 40.0	66.3 ± 23.1	65.3 ± 38.5	0.327
Total protein (g/L)	67.8 ± 7.2	69.7 ± 6.0	68.8 ± 6.3	68.0 ± 7.8	67.6 ± 6.5	65.2 ± 8.2	< 0.001

Abbreviations: CRC = colorectal cancer, PLR = platelet lymphocyte ratio, BMI = body mass index, DM = diabetes mellitus, CEA = carcinoembryonic antigen

The median preoperative PLR was 169.1. By applying receiver operating curve analysis, the optimal cut-off value for the PLR was 157.8 both for OS and for DFS. The cut off values for categorization of PLR into quintiles were 100, 120, 160 and 220. A higher PLR was significantly associated with lower values of BMI at diagnosis (*P* < 0.01). Patients in PLR quintiles 5 were significantly associated with higher tumor stages, particularly stage IV disease. The tumors were also more likely to be associated with poor outcome predictors such as vascular invasion, total protein (*P* < 0.05 for all measurements). There were no statistically significant differences in other clinic-pathological factors.

### The prognosis impact of the PLR on overall and disease-free survival

**Figure 1:**
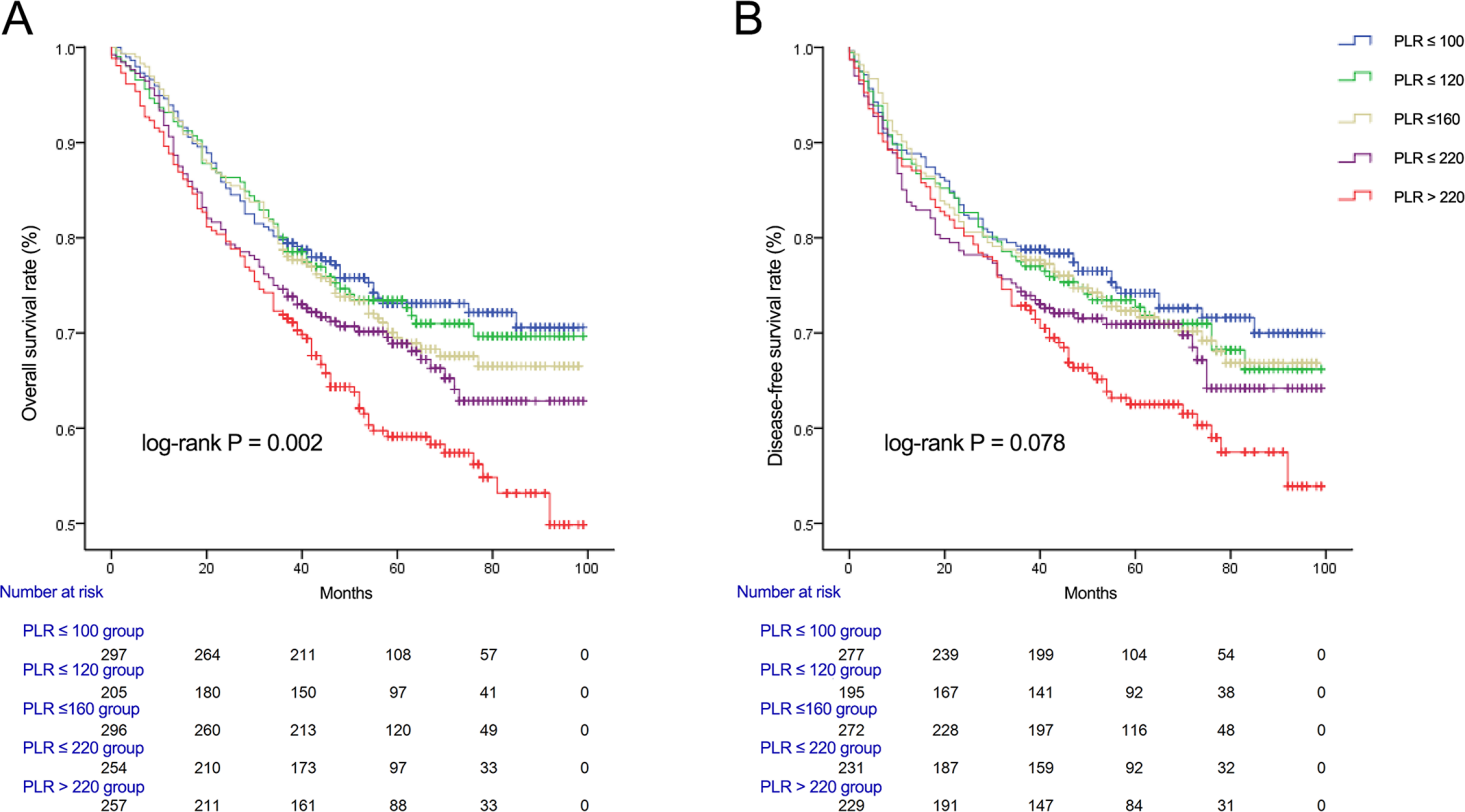
Kaplan-Meier survival curves showing overall survival (A) and disease-free survival (B) stratified by quintiles of PLR in colorectal cancer patients

The mean follow-up time was 59.6 months. Kaplan-Meier analysis of OS and DFS showed progressively worse OS with each PLR quintile (*P* = 0.002; Figure 1A). Patients with high DFS more likely linked with the low PLR, although the difference in DFS was not statistically significant (*P* = 0.078; Figure 1B).

### Cox analyses of survival associated with PLR

Unadjusted univariable Cox proportional hazard models were used to identify variables associated with OS and DFS and the results were presented in Table 2. The patients in highest quintile of PLR had 70% increase in hazard death and 52% increased hazard of having recurrence of disease compared with the first quintile (hazard ratio (HR) = 1.701; 95% confidence interval (CI) 1.267–2.282, *P* < 0.001 and HR =1.522; 95% CI 1.114–2.080, *P* = 0.008, respectively). Gender, age, BMI, tumor TNM stage, tumor differentiation, the presence of vascular invasion, total protein and CEA were also significantly associated with the hazard of death in the univariate analysis (*P* < 0.05 for all measurements). In the multivariate analysis, PLR remained significantly associated with OS (HR =1.511; 95% CI 1.103–2.070, *P* = 0.010). However, BMI and the presence of vascular invasion were not associated with OS. In the multivariate Cox analysis of DFS, gender, TNM stage, tumor differentiation and total protein were independent predictive risk factors for the prognosis of patients after adjustment for PLR, vascular invasion, and CEA (*P* < 0.05 for all measurements, Table 2).

**Table 2:** Cox proportional hazards regression models of risk factors associated with overall and disease-free survival among CRC patients

	Overall Survival						Disease–free Survival					
	Univariable			Multivariable			Univariable			Multivariable		
	HR	95% CI	*P*	HR	95% CI	*P*	HR	95% CI	*P*	HR	95% CI	*P*
Gender(male vs female)	1.389	1.133–1.704	0.002	1.331	1.078–1.645	0.008	1.372	1.106–1.701	0.004	1.307	1.046–1.632	0.018
Age	1.011	1.003–1.019	0.009	1.011	1.003–1.020	0.007	1.007	0.998–1.015	0.111			
BMI (kg/m2)	0.968	0.939–0.998	0.035				0.984	0.954–1.016	0.328			
PLR(continuous)	1.001	1.000–1.001	0.040	1.001	1.000–1.002	0.034	1.001	1.000–1.001	0.100	1.001	1.000–1.002	0.078
Quintile 1	1.000	–	–	1.000	–	–	1.000	–	–	1.000	–	–
Quintile 2	1.050	0.746–1.479	0.778	0.823	0.578–1.173	0.281	1.110	0.784–1.574	0.557	0.906	0.629–1.303	0.593
Quintile 3	1.146	0.844–1.554	0.383	1.038	0.840–1.605	0.816	1.108	0.805–1.525	0.528	1.047	0.752–1.459	0.785
Quintile 4	1.318	0.966–1.797	0.081	1.161	0.840–1.605	0.365	1.246	0.899–1.728	0.186	1.126	0.800–1.584	0.497
Quintile 5	1.701	1.267–2.282	< 0.001	1.511	1.103–2.070	0.010	1.522	1.114–2.080	0.008	1.356	0.970–1.896	0.075
TNM Staging	0.220	0.176–0.275	< 0.001	0.243	0.192–0.307	< 0.001	0.322	0.260–0.398	< 0.001	0.352	0.280–0.442	< 0.001
Stage I	1.000	–	–				1.000	–	–			
Stage II	1.009	0.6640–1.533	0.967				1.022	0.805–1.484	0.907			
Stage III	3.438	2.356–5.016	< 0.001				3.119	2.220–4.381	< 0.001			
Stage IV	17.431	11.575–26.248	< 0.001				11.790	4.199–33.102	< 0.001			
Differentiation	0.580	0.474–0.711	< 0.001	0.752	0.607–0.932	0.009	0.604	0.486–0.750	< 0.001	0.735	0.584–0.926	0.009
Vascular invasion	0.528	0.416–0.669	< 0.001				0.608	0.465–0.795	< 0.001			
Total protein	0.980	0.967–0.993	0.002	0.984	0.970–0.998	0.022	0.978	0.965–0.992	0.002	0.978	0.964–0.993	0.003
CEA	1.000	1.000–1.001	< 0.001	1.001	1.001–1.001	< 0.001	1.000	1.000–1.001	< 0.001	1.000	1.000–1.001	0.309
Creatinine	1.000	0.997–1.003	0.958				1.001	0.998–1.004	0.536			
DM	0.980	0.707–1.358	0.905				0.948	0.672–1.338	0.763			
Hypertension	0.873	0.707–1.078	0.206				0.837	0.670–1.046	0.117			
Obesity	0.938	0.717–1.227	0.641				1.159	0.876–1.535	0.301			

Abbreviations: CRC = colorectal cancer, PLR = platelet lymphocyte ratio, BMI = body mass index, CEA = carcinoembryonic antigen, DM = diabetes mellitus, HR = hazard ratio, CI = confidence interval.

Based on the optimal cut-off values and those applied in previous studies [14, 15, 17, 18] we chose values of 150, 185, 220 and 300 for the dichotomous analysis. Sensitivity analyses for the PLR with different cut of values returned qualitatively similar results (Table 3). In a adjusted multivariable analysis, CRC patients with the higher level of PLR were significantly associated with a higher risk of mortality compared with their counterparts with the lower PLR using the different cut-off values of 150, 185 and 220 (*P* = 0.002, *P* = 0.014, *P =* 0.001, respectively). However, by applying a cut-off of 300, a high PLR was not significantly associated with the risk of mortality (*P* = 0.055). For DFS, using the cut-off values of 150 and 220, a high PLR was significantly associated with the risk of disease recurrence (*P* = 0.033, *P* = 0.024, respectively) (Table 3). Sensitivity analyses using this different set of the PLR quintiles (cut-off values 150, 185, 220, 300) did not change the main results (Supplementary Figure 1).

**Table 3:** Association between PLR and mortality in CRC patients applying different cutoff values

PLR	Overall Survival					Disease–free Survival				
	Total	Univariable		Multivariable*		Total	Univariable		Multivariable*	
		HR 95% CI	*P*	HR 95% CI	*P*		HR 95% CI	*P*	HR 95% CI	*P*
≤ 150	747	1.000	0.001	1.000	0.002	700	1.000	0.024	1.000	0.033
> 150	567	1.387 (1.143–1.682)		1.378 (1.123–1.691)		513	1.267 (1.032–1.556)		1.267 (1.020–1.575)	
≤ 185	932	1.000	0.002	1.000	0.014	816	1.000	0.022	1.000	0.069
> 185	382	1.380 (1.126–1.691)		1.314 (1.056–1.634)		316	1.289 (1.037–1.603)		1.241 (0.983–1.567)	
≤ 220	1054	1.000	0.001	1.000	0.001	981	1.000	0.010	1.000	0.024
> 220	260	1.511 (1.211–1.887)		1.492 (1.174–1.896)		232	1.372 (1.079–1.745)		1.346 (1.040–1.742)	
≤ 300	1207	1.000	0.179	1.000	0.055	1135	1.000	0.397	1.000	0.155
> 300	107	1.254 (0.902–1.744)		1.408 (0.993–1.995)		93	1.167 (0.816–1.669)		1.312 (0.903–1.906)	

Abbreviations: CRC = colorectal cancer, PLR = platelet lymphocyte ratio, HR = hazard ratio, CI = confidence interval.

* Hazard ratios for PLR were derived using Cox regression adjusted for age at diagnosis, gender, TNM stages, tumor differentiation, the presence of vascular invasion, total protein, CEA.

### Subgroup analyses associated with PLR

**Figure 2:**
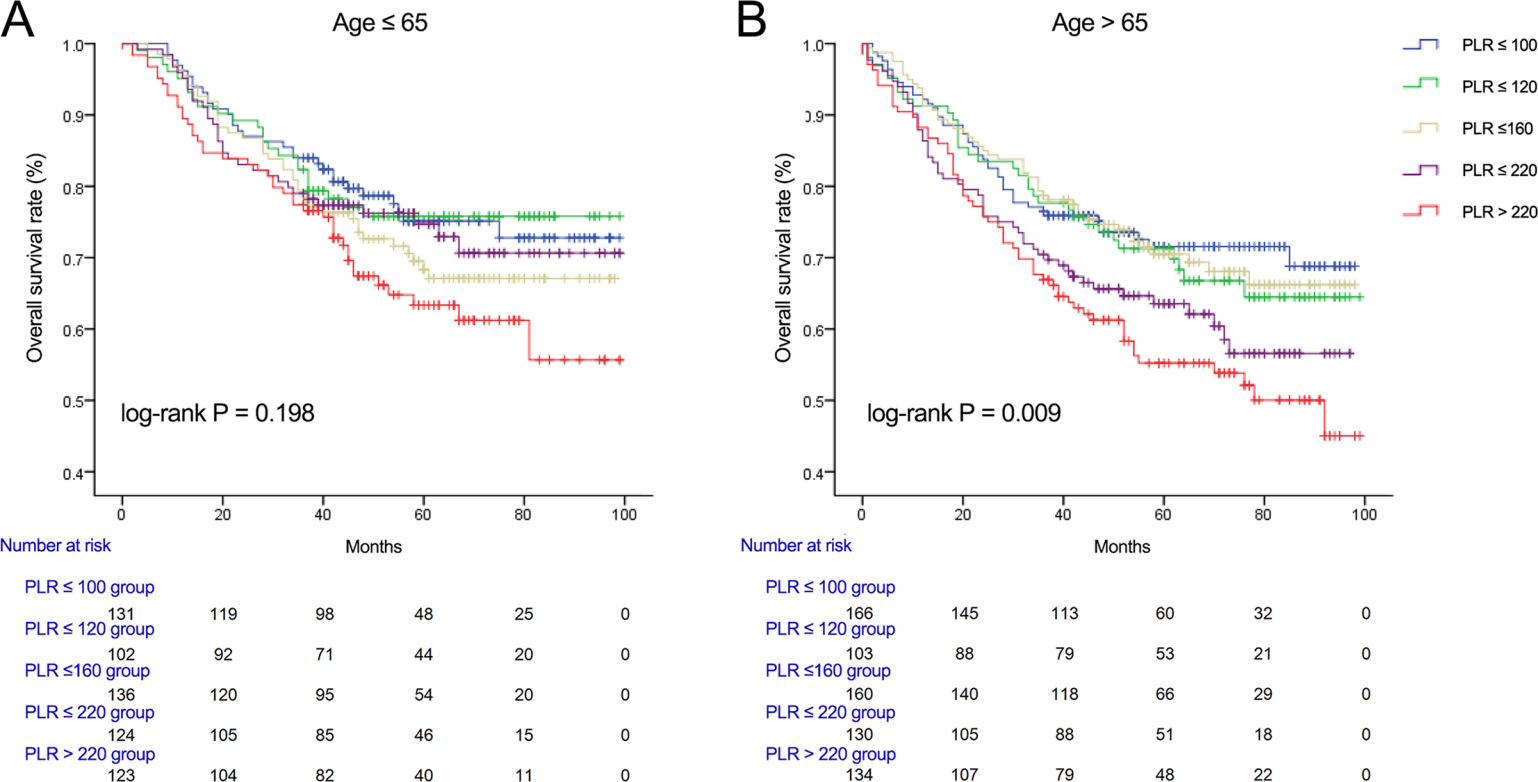
Overall survival of CRC patients stratified by quintiles of PLR according to (A) young age (≤ 65) and (B) old age (> 65)

**Figure 3:**
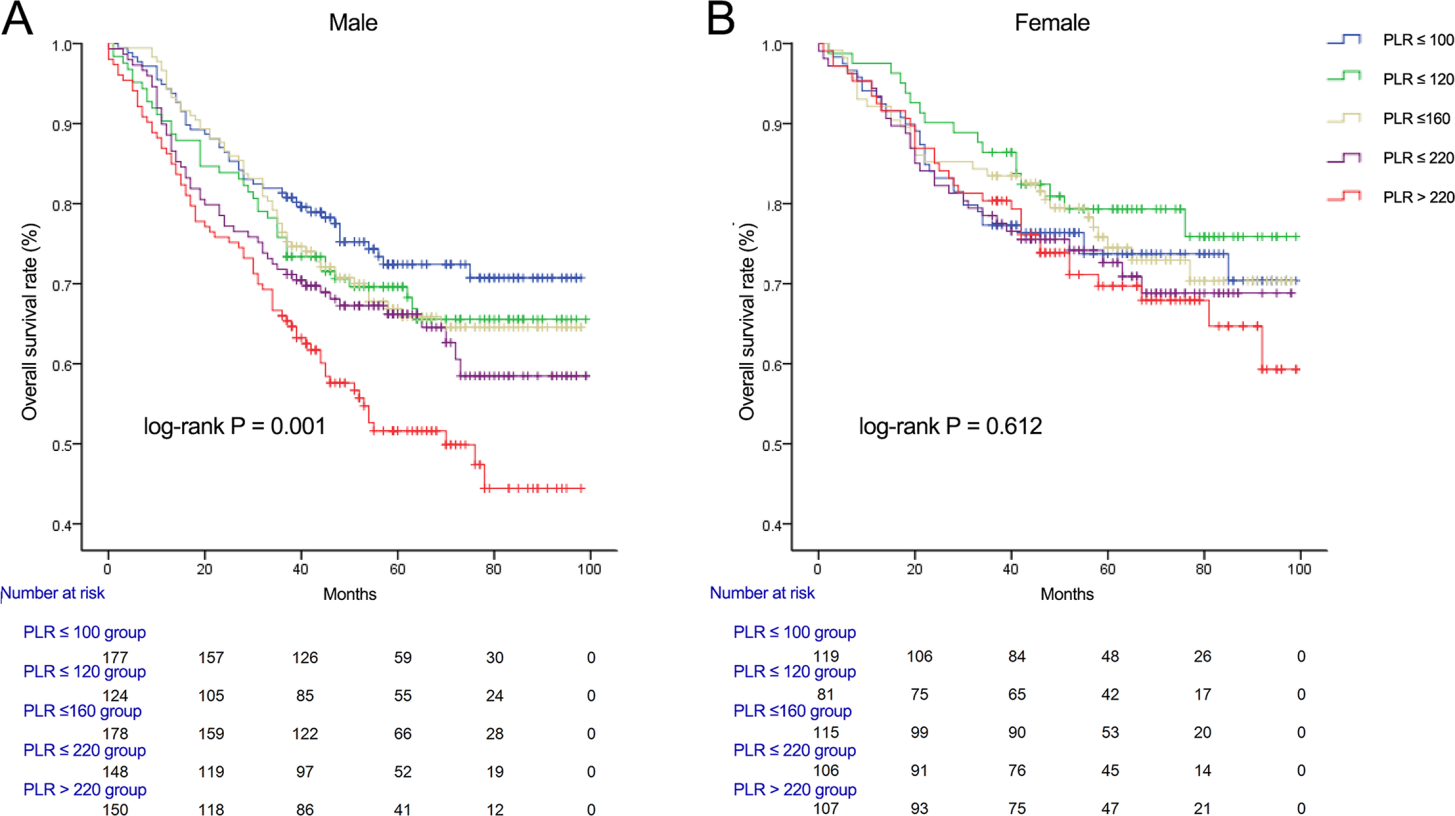
Overall survival of CRC patients stratified by quintiles of PLR according to male (A) and female (B)

**Figure 4:**
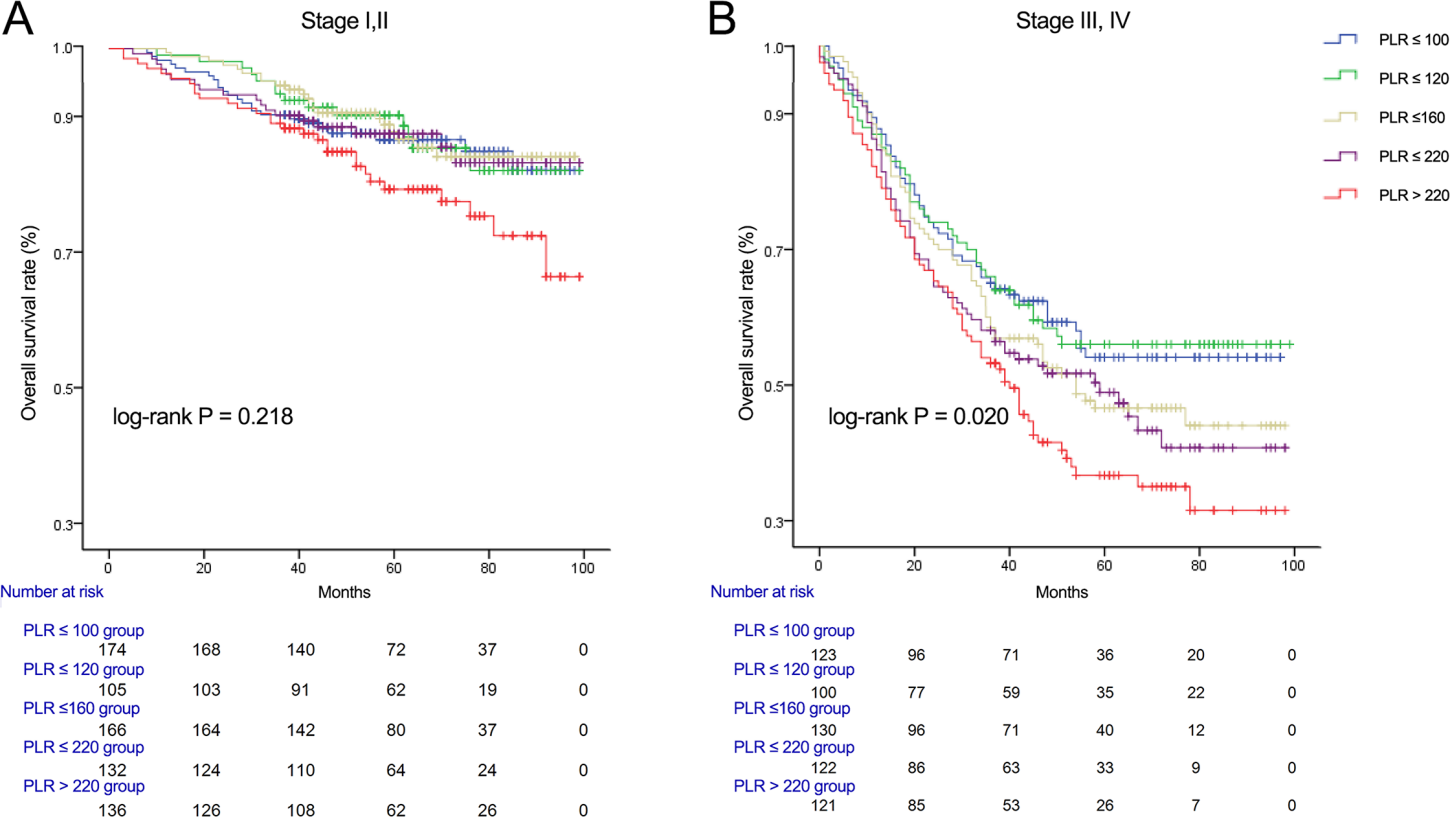
Overall survival of CRC patients stratified by quintiles of PLR according to tumor stage I, II (A) and tumor stage III,IV (B)

**Figure 5:**
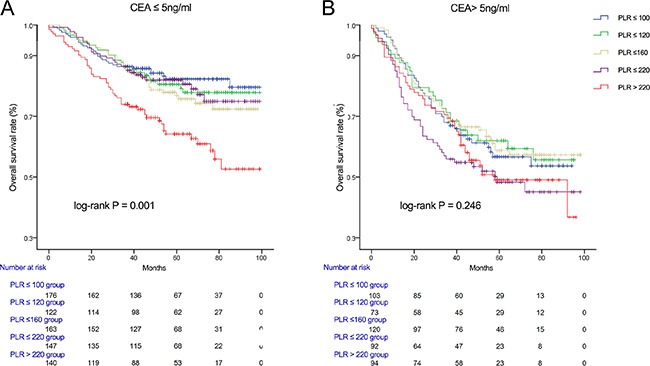
Overall survival of CRC patients stratified by quintiles of PLR according to CEA ≤ 5 ng/ml (A) and CEA > 5 ng/ml (B)

**Figure 6:**
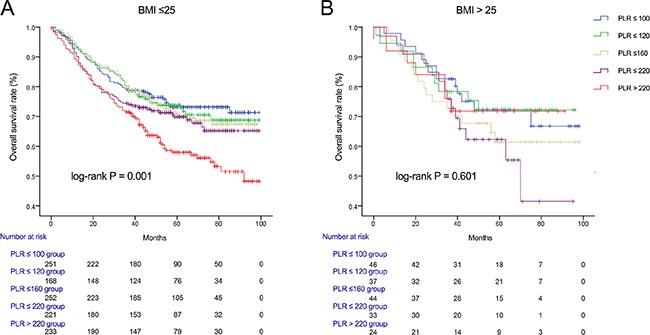
Overall survival of CRC patients stratified by quintiles of PLR according to BMI ≤ 25 (A) and BMI > 25 (B)

**Figure 7:**
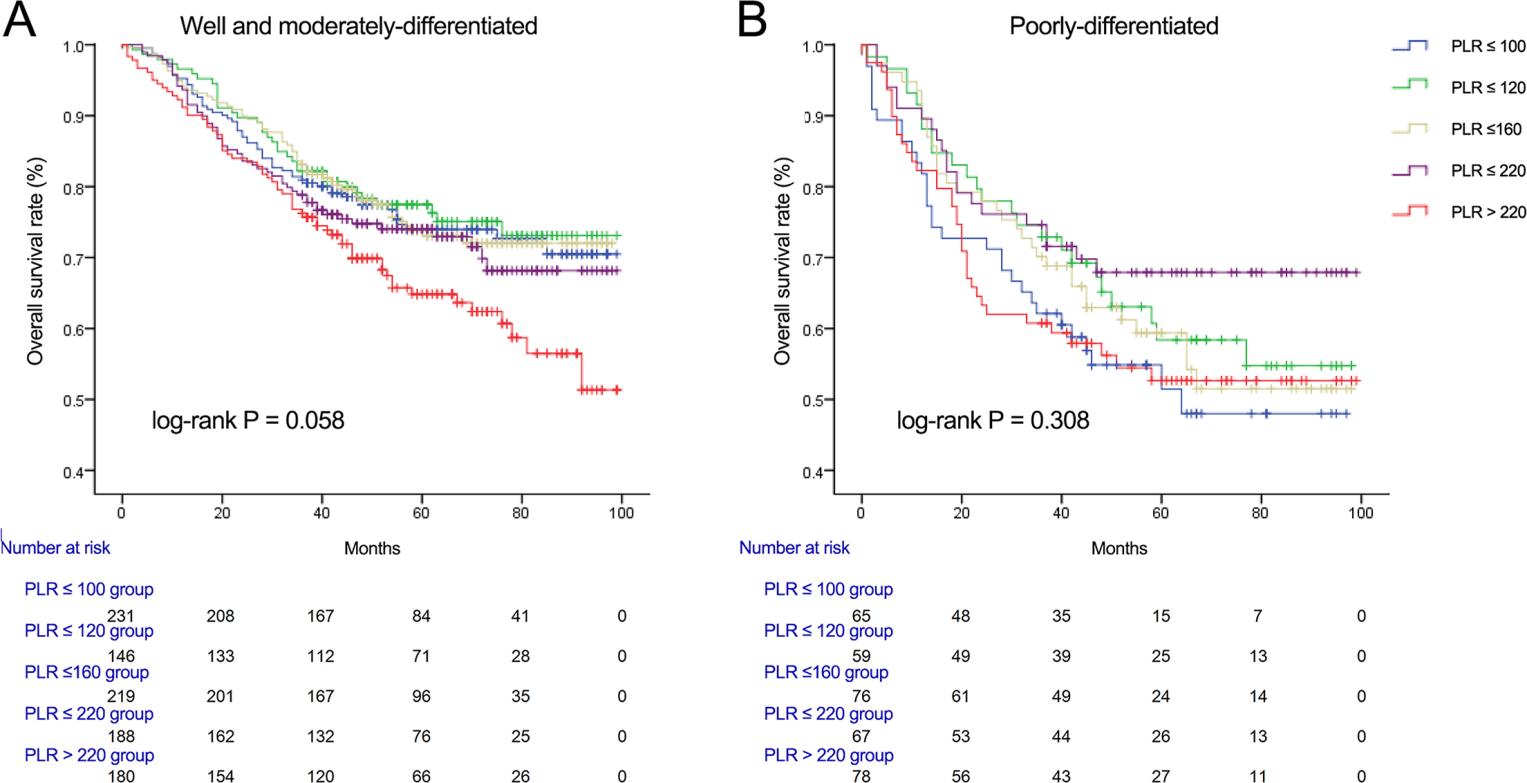
Overall survival of CRC patients stratified by quintiles of PLR according to well and moderate differentiation (A) and poor differentiation (B)

We used Kaplan-Meier methodology to examine the impact of PLR on OS in patients stratified by age, BMI, tumor differentiation, gender, tumor stage and CEA, PLR quintiles were significantly associated with poor survival in older (> 65 years) (Figure 2B) male (Figure 3A) patients with advanced tumor TNM stage (III and IV) (Figure 4B), normal range of CEA (≤ 5ng/ml) (Figure 5A) and BMI (≤ 25) (Figure 6A) (*P* < 0.01 for all measurements). When stratified by tumor differentiation, PLR was closely associated with poor survival in patients with well and moderate differentiation, although not significantly (*P* = 0.058) (Figure 7A).

When compared with the quintile representing the lowest value of PLR, CRC patients in the highest quintile of the PLR were also found to have substantially lower survival rates in several aspects, such as male (HR = 2.116, 95% CI 1.461–3.064), BMI (≤ 25) (HR = 1.785, 95% CI 1.302 – 2.445), tumor stage (III and IV) (HR = 1.657, 95% CI 1.166 – 2.354), tumor differentiation (well and moderately) (HR = 1.511, 95% CI 1.060–2.152), CEA (≤ 5ng/ml) (HR = 2.403, 95% CI 1.533–3.768), irrespective of age (≥ 65) (HR = 1.776, 95% CI 1.213–2.601) or young age (< 65) (HR = 1.622, 95% CI 1.022–2.574) (Table 4).

**Table 4:** Cox proportional hazard regression analysis of OS in CRC patients stratified by clinic-pathological variables according to PLR quintiles

Characteristic	Total	PLR quintiles					*P* value trend^a^
		Quintile 1	Quintile 2	Quintile 3	Quintile 4	Quintile 5	
Age (years)							
> 65	697	1.00	1.112 (0.711–1.740)	1.046 (0.698–1.567)	1.476 (0.991–2.199)	1.776 (1.213–2.601)^b^	0.011
≤ 65	617	1.00	1.001 (0.588–1.706)	1.294 (0.812–2.063)	1.145 (0.699–1.877)	1.622 (1.022–2.574)^b^	0.218
Gender							
Male	785	1.00	1.260 (0.825–1.926)	1.255 (0.852–1.848)	1.476 (0.994–2.191)	2.116 (1.461–3.064)^b^	0.001
Female	529	1.00	0.756 (0.418–1.366)	0.942 (0.568–1.563)	1.109 (0.671–1.833)	1.182 (0.724–1.930)	0.619
BMI (kg/m2)							
> 25	185	1.00	0.934 (0.409–2.132)	1.377 (0.662–2.863)	1.622 (0.761–3.453)	1.074 (0.428–2.691)	0.610
≤ 25	1129	1.00	1.076 (0.738–1.568)	1.108 (0.792–1.550)	1.272 (0.905–1.788)	1.785 (1.302–2.445)^b^	0.001
TNM Staging							
Stage I, II	713	1.00	0.910 (0.470–1.759)	0.870 (0.484–1.563)	0.981 (0.532–1.807)	1.560 (0.908–2.679)	0.229
Stage III, IV	601	1.00	0.982 (0.657–1.468)	1.238 (0.864–1.773)	1.329 (0.925–1.911)	1.657 (1.166–2.354)^b^	0.005
Differentiation							
Well and Moderately	966	1.00	0.913 (0.600–1.388)	0.985 (0.682–1.422)	1.129 (0.777–1.642)	1.511 (1.060–2.152)^b^	0.062
Poorly,	348	1.00	0.738 (0.433–1.258)	0.807 (0.494–1.318)	0.582 (0.334–1.013)	0.946 (0.585–1.530)	0.320
CEA (ng/ml)							
> 5	484	1.000	0.930 (0.580–1.489)	0.917 (0.607–1.384)	1.363 (0.901–2.062)	1.210 (0.798–1.834)	0.255
≤ 5	751	1.000	1.145 (0.673–1.947)	1.347 (0.834–2.173)	1.193 (0.719–1.979)	2.403 (1.533–3.768)^b^	< 0.001

Abbreviations: PLR = platelet lymphocyte ratio, OS = overall survival, BMI = body mass index, CEA = carcinoembryonic antigen, CI = confidence interval

a P for trend is computed by entering the quintiles as discontinuous terms in the Cox model

b Statistically significant (*P* < 0.05)

## DISCUSSION

In this study, a higher preoperative PLR was significantly and independently associated with higher mortality in patients with CRC (*P* = 0.002), with evidence that a cut off value could be derived from the selected. Similarly, the PLR showed close association with DFS, although not significant (*P* = 0.078). The results remained robust when using different cut off values and on analysis of the subgroups stratified by clinic-pathological factors, especially when comparing the highest quintile of PLR with the lowest quintile. The PLR showed a close relationship with not only tumor related characteristics, such as tumor stage and the serum level of CEA, but also nutritional status, such as total protein and BMI, which also reflected cachexia due to hypercytokinemia resulting from tumor progression [19]. Among the CRC patients stratified by clinical-pathological factors, the results from multivariate analysis indicated that the PLR was associated with OS, along with tumor-related factors, such as tumor stage, the presence of vascular invasion and CEA. It could be concluded, therefore, that the PLR have potential utility in predicting the mortality of patients with CRC.

Recent studies have demonstrated that the elevated PLR has a relationship with adverse postoperative survival in patients with several types of cancer, such as pancreatic [20], ovarian [21], gastric [22], prostate [23], esophagus [24] and colorectal cancer [13–15]. Previously, two meta-analyses of the patients with solid tumors have demonstrated that a high PLR was associated with a worse OS in various solid tumors including CRC [25, 26]. Although current evidence supporting a role for the PLR as a prognostic indicator for patients with CRC has been relatively sparse and the power of predictive survivability less than other inflammation-based scores, such as Glasgow Prognostic Score and value of neutrophil-to-lymphocyte ratio [16, 18, 27, 28], our results provide compelling evidence that the PLR is also an adverse prognostic predictors in CRC.

In the most circumstances, the optimal cut-off values for prognostic factors are selected by validating previously established cut-off values from other clinical studies. When analyzing the cut-off values of PLR, previous studies have mainly used dichotomous categorization [13, 15, 18, 29] and trichotomy cut-off [14, 27] and we therefore further performed sensitivity analyses by using dichotomous categorization. The results indicated that a cut-off of value of 220 for PLR, which was also the cut-off value for CRC patients in the highest quintile in this study, was able to discriminate between those with a higher risk of mortality and those with a lower risk, replicating the same findings as the risk of recurrence of the disease.

Although most of the previous studies showed that PLR had higher HRs for mortality than the observed HR in current study [13–15], some recent studies did not find an association between PLR and OS, or DFS [16, 30]. One recent study which utilized a cut-off value of 300 [18], has shown that high PLR was not associated with worse OS and DFS in multivariable models, which is in agreement with our findings. One possible explanation is that the higher the cut-off value chosen, the smaller numbers of patients are enrolled in clinical practice and in the current study, there were only 8.1% of patients in the PLR group of greater than 300. In the subgroup analyses of the PLR, the impact of PLR on OS was not significantly associated with poor survival by age (≤ 65) and sex and one possible explanation is that the mean 59.6 months period of follow-up was not long enough to show a statistically significant effect. Another explanation for the results from the subgroup specified by age, is that the CRC patients aged ≤ 65 would have longer life expectancy than patients aged < 65 after surgical treatment. According to recent cancer statistics in China, male CRC patients had a higher estimated mortality than females [31] and 5-year overall and a multivariate proportional hazard model [32] showed that cancer-specific survival of CRC was significantly higher in women than in men. This may suggest that longer follow-up periods may be required to see significant outcomes in both women and men.

The specific mechanisms by which the PLR influences the prognosis for patients with CRC remains incompletely understood. It is well known that malignant solid tumors commonly induce a hypercoagulable state, which may gradually lead to thrombocytosis [33, 34] and this has been considered a negative prognostic factor for patients with solid cancers [35]. The pro-inflammatory cytokine IL-6 has an important role in the onset of reactive thrombocytosis [36]. Similarly, IL-6 has a cell-proliferative effect, promoting the differentiation of megakaryocytes to platelets in the bone marrow [37]. Peripheral platelets, lymphocytes or their ratio are thought to be indicators of the inflammatory process induced by cancer cells and a high level of platelets may promote tumor growth by increasing angiogenesis through production of vascular endothelial growth factor which has been shown to be associated with disease prognosis in patients with various cancers [38]. Lymphocytes, however, play a vital role in cancer immune surveillance and suppress a tumor maturation [39] and a decreased concentration of intratumoral CD8^+^ cytotoxic lymphocytes has been strongly associated with disease prognosis in patients with colorectal cancer [40].

In summary, our current study suggests that the preoperative PLR is an independent prognostic factor in patients with CRC. Future studies are required to further validate the prognostic and predictive values of PLR.

## METHODS

### Patients

A total of 1314 patients who underwent surgical resection for colorectal adenocarcinoma between April 2005 and April 2011 at the First Affiliated Hospital of Wenzhou Medical University, were enrolled in this study. Patients who had clinical evidence of infection, hematological disease, enterobrosis, intestinal obstruction and received neoadjuvant therapy were excluded. All patients exceeded 18 years of age. The study was approved by the Ethics Committee of the First Affiliated Hospital of Wenzhou Medical University and written informed consents were obtained from every patient.

### Clinical-pathological and laboratory data

Demographic, preoperative laboratory and pathologic data of all patients were collected from electronic medical records and reviewed. Detailed clinical data was conducted within 2 weeks before operation. Preoperative blood values including white cell, neutrophil, lymphocyte, monocyte and platelet counts were collected from a routine blood test before surgical operation. PLR was calculated as the absolute platelet count divided by absolute lymphocyte count and was determined using a Hitachi 7600 chemistry analyzer (Hitachi, Tokyo, Japan) with the kinetic method. Body mass index (BMI) was calculated as weight in kilograms divided by height in meters squared (kg/m^2^). Subjects were defined as obese if BMI was greater than or equal to 25 kg/m^2^.

Patients with CRC were treated primarily by surgical resection with adjuvant chemotherapy for node-positive patients and node-negative patients with adverse pathological features according to the National Comprehensive Cancer Network guidelines. Tumor staging of CRC was performed according to the sixth edition of the American Joint Committee on Cancer staging manual. Information regarding tumor location, TNM staging and histological differentiation of tumors and vascular invasion was collected from pathological and colonoscopic sample analyses.

### Follow-up data

Patients were followed up in a post-operative outpatient schedule every 3–6 months for 2 years, every 6 months thereafter for a total of 5 years and every 1 year thereafter. Colonoscopy and computed tomography (CT) were obtained at post-operative follow-up appointments in addition to blood analysis including carcinoembryonic antigen (CEA). Tumor recurrence such as suggested by elevated CEA, abnormal findings on colonscopy or the CT scan was defined as an earlier follow-up event. Information on death was obtained either from the patient's social security death index, outpatient medical records, or notifications from the family of the deceased. The deadline of follow-up time was June 1, 2014. OS was calculated from the date of surgery to the date of death or the date of last follow-up. Disease-free survival (DFS) was calculated as the time from the date of surgery to the time of recurrence or date of last follow-up.

### Statistical analysis

Continuous variables were tested for normality by using the Kolmogorov-Smirnov test. Continuous data with a normal distribution were expressed as the mean ± standard deviation and compared using a standard *t* test. Otherwise, continuous data with non-normal distribution were compared using the Wilcoxon rank-sum test. Categorical variables were expressed as percentage and compared using the Chi-square test or Fisher's exact test as appropriate. Based on the distribution of PLR and the size of the study population, patients were stratified into quintiles of the PLR (quintile 1, quintile 2, quintile 3, quintile 4, quintile 5). The demographic and clinic-pathological characteristics were compared between the quintiles. The optimal cut-off levels for PLR were calculated by applying receiver operating curve analysis for the dichotomous categorization. Kaplan-Meier survival curves with log-rank tests and Cox proportional hazard regression analyses, recording patients at the time of last follow-up visit, were used to compare the OS and DFS rates. Variables with *P* ≤ 0.1 in the univariate Cox regression analysis were progressed to a multivariate analysis using forward stepwise selection. All *P* values were two sided and a *P* value < 0.05 was considered to be statistically significant. Statistical analysis was performed using SPSS version 19.0 software (SPSS, Chicago, IL, USA) and Med Calc version 13.0.0.0 (Med Calc Software, Mariakerke, Belgium).

## SUPPLEMENTARY MATERIALS FIGURE


