# Supplementary material for: Preoperative platelet to lymphocyte ratio is a valuable prognostic biomarker in patients with colorectal cancer
Source: Oncotarget. 2016 Mar 24;7(18):25516–27. doi: 10.18632/oncotarget.8334 (PMC5041922; doi:10.18632/oncotarget.8334)
Supplement: Supplementary file 1 [file oncotarget-07-25516-s001.pdf]

Preoperative platelet to lymphocyte ratio is a valuable prognostic biomarker in patients with colorectal cancer

Supplementary Materials

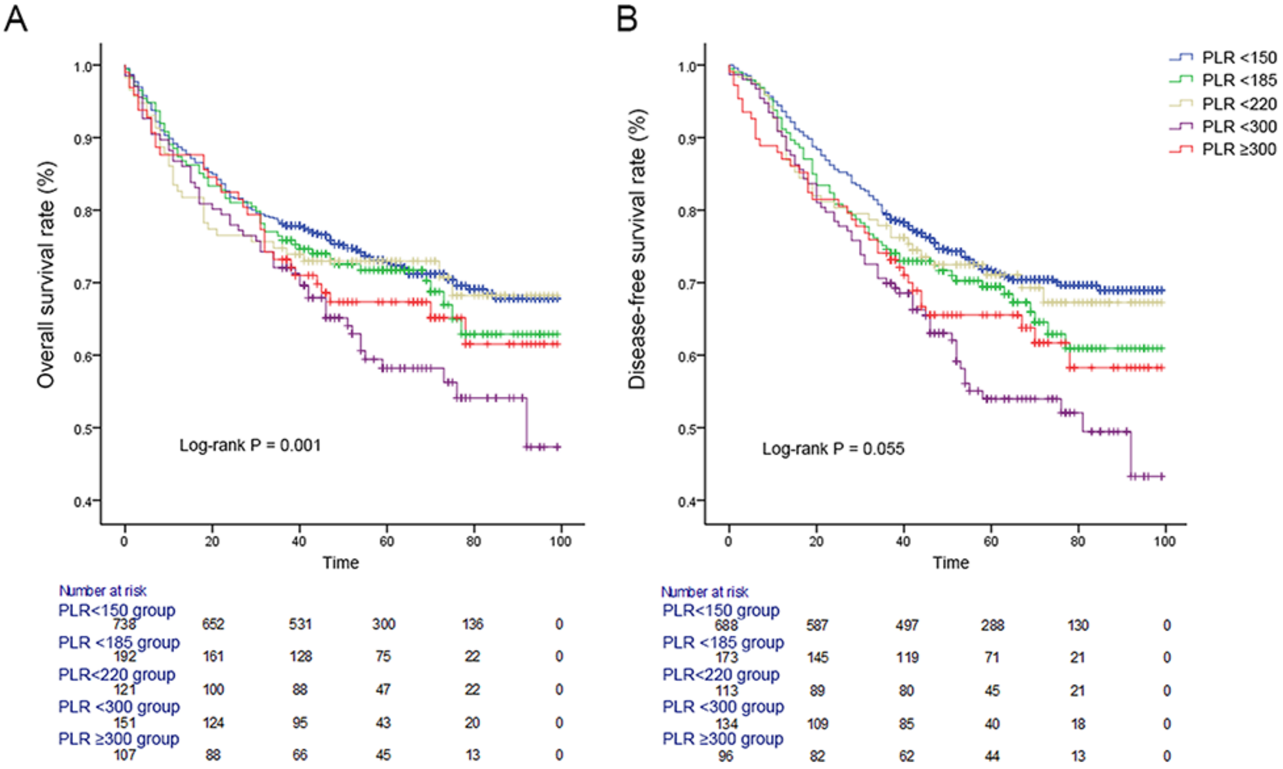

Supplementary Figure S1: Kaplan-Meier survival curves showing overall survival (A) and disease-free survival (B) stratified by quintiles of PLR in colorectal cancer patients (cut-off values 150, 185, 220, 300 respectively).
